# Dynamical modeling of multi-scale variability in neuronal competition

**DOI:** 10.1038/s42003-019-0555-7

**Published:** 2019-08-23

**Authors:** Benjamin P. Cohen, Carson C. Chow, Shashaank Vattikuti

**Affiliations:** Mathematical Biology Section, Laboratory of Biological Modeling, National Institutes of Diabetes and Digestive and Kidney Disease, National Institutes of Health, Bethesda, MD USA

**Keywords:** Computational biophysics, Perception, Biophysical models

## Abstract

Variability is observed at multiple-scales in the brain and ubiquitous in perception. However, the nature of perceptual variability is an open question. We focus on variability during perceptual rivalry, a form of neuronal competition. Rivalry provides a window into neural processing since activity in many brain areas is correlated to the alternating perception rather than a constant ambiguous stimulus. It exhibits robust properties at multiple scales including conscious awareness and neuron dynamics. The prevalent theory for spiking variability is called the balanced state; whereas, the source of perceptual variability is unknown. Here we show that a single biophysical circuit model, satisfying certain mutual inhibition architectures, can explain spiking and perceptual variability during rivalry. These models adhere to a broad set of strict experimental constraints at multiple scales. As we show, the models predict how spiking and perceptual variability changes with stimulus conditions.

## Abstract (web summary)

Benjamin P Cohen, Carson C Chow, and Shashaank Vattikuti show that dynamical mutual inhibition models can explain variability during neuronal competition at two scales: neuronal spiking activity and perceptual rivalry variability. These models make predictions for how spiking and perceptual variability will change with stimulus conditions.

## Introduction

Variability is observed at multiple scales in the brain. At the microscopic level, ion channels and synapses are subject to random effects of molecular discreteness^1,2^. Neocortical neurons fire stochastically and follow Poisson- or super-Poisson-like statistics^3–5^. At the cognitive level, variability is observed in behavior and perception. It is not clear how the variability at one scale is related to the variability at another scale. Variability at a small scale could induce variability at a larger scale or be averaged away and not be relevant. The nontriviality in the connection between microscopic and macroscopic variability played out previously in the attempt to explain spiking variability. While it was well known that ion channels and synapses are subject to small number biochemical variability, in vitro neuron spiking was found to be quite reliable when driven^6,7^. A resolution to this paradox invokes an attractor state with balanced excitatory and inhibitory synaptic inputs that yield a net input to neurons close to threshold so that fluctuations in the inputs drive spiking. Irregular spiking emerges robustly when the network settles into a chaotic attractor termed the *balanced state*^8,9^. In this case the variability is due to a deterministic albeit chaotic process. Here, we examine and quantify the relationship between spiking variability and perceptual variability.

Variability is ubiquitous in perception. It may serve a functional role for optimizing foraging^10^, learning patterns such as songs^11,12^, and for producing unpredictable trajectories while evading predators^13,14^. It may also help to arbitrate ambiguous circumstances such as that posed in the paradox of Buridan’s ass who, equally hungry and thirsty, is placed precisely midway between a stack of hay and a pail of water and cannot decide. Perceptual variability can break this symmetry and release the ass from its fatal dilemma. The nature and source of perceptual variability is an open question. Although noise from the environment is important, perceptual variability is still observed when the stimulus conditions are controlled^2,15^ and even when the eyes are paralyzed in a visual task (Leon Lack personal communication). Given that neuronal spiking is correlated with perception, spiking variability is a compelling etiology for the perceptual variability^5,16^. However, the precise mechanistic relationship between spiking and perceptual variability is unknown.

We focus on perceptual variability during neuronal competition and particularly perceptual rivalry. Neuronal competition is a ubiquitous property of the brain, playing a role in cognitive models of forced choice decision making^17,18^, flanker-suppressor tasks^19^, short-term memory^20,21^, and other computations^22,23^. Perceptual rivalry is a form of dynamic neuronal competition where the perception alternates between plausible interpretations given a fixed ambiguous stimulus and neural activity (in many brain regions) is correlated with the perception^24^. It is found in many visual contexts such as binocular rivalry, Necker cube, face-vase illusion, and motion-induced blindness, and also been reported for almost all sensory modalities^25^. The percept durations in rivalry obey a gamma-like distribution with coefficient of variation and skewness that is tightly constrained. This distribution is robust across many conditions, suggestive of intrinsic variability. It is found for both vision and audition^26^, across species^27^, and across a variety of visual stimulus conditions^28^. Despite the pervasiveness of the percept distribution there is no biophysical explanation for these statistics.

There is a long history of modeling neuronal competition with biophysically constrained cortical circuits^20,29,30^. For example, a circuit with lateral or mutual inhibition can exhibit *winner-take-all dynamics (WTA)* where a pool of neurons tuned to a percept suppresses the remaining pools^19,21,31^. With the inclusion of a fatigue mechanism, rivalrous alternations can arise from the WTA state^19,21,29,32^. However, rivalry is a challenge for quantitative modeling because of the many experimental constraints. Some models match the observed perceptual variability without adding noise but they fail to account for realistic spiking statistics (irregular, asynchronous spiking)^29^. Competition-like dynamics with variability have been demonstrated in deterministic balanced state networks but they have not been rigorously tested against perceptual constraints. An unstructured randomly connected network can produce alternating activity levels between two pools when receiving different fluctuating external inputs^33^. With structured connections and constant input, these models can exhibit temporary up-states or winnerless competition with balanced dynamics^15,34–36^. It has also been found that asymmetric activity levels in a balanced state can be achieved in mutual inhibition networks with a mechanism distinct from WTA^37^. A WTA network with mutual inhibition and high spiking variability has been invoked to explain choice probabilities in a perceptual decision-making task^18^ but external noise contributed to the spiking variability. Thus, it remains to be seen whether balanced state and rivalry dynamics can coexist.

It is not clear that the balanced state can coexist with rivalry *prima facie*. Balanced state theory is predicated on a dynamic balance between excitation and inhibition. Rivalry strongly depends on imbalanced connections (e.g., mutual inhibition) between percept-encoding neuronal pools to produce a WTA state, and on a fatigue mechanism (e.g., spike-frequency adaption, synaptic depression), which is important for alternations. Neuronal adaptation has mixed effects. It can either aid irregularity by homogenizing synaptic inputs and facilitating a balanced state^38,^ or increase synchrony^39,40^ and thus be antagonistic to commonly observed asynchrony. Finally, matching variability at one scale does not ensure matching at another scale without invoking additional mechanisms. For example, spiking variability may be too large or too small in magnitude or have no impact for perceptual variability. Thus, it is not a priori obvious how rivalry and all of its constraints can include biophysical spiking and whether the balanced state is a viable solution.

Here we show that unstructured networks cannot explain rivalry but networks with structured mutual inhibition, adaptation, and network-induced biophysical spiking statistics can. We deploy the balanced state theory to show that this network breaks global balance; the dominant pool is balanced but the suppressed pool is not although it fires irregularly due to random input from the dominant pool. The mechanism is also robust to connection architectures. In summary, we provide a self-consistent mechanism for spiking and perceptual variability.

## Results

### Unstructured network does not capture rivalry

**Fig. 1:**
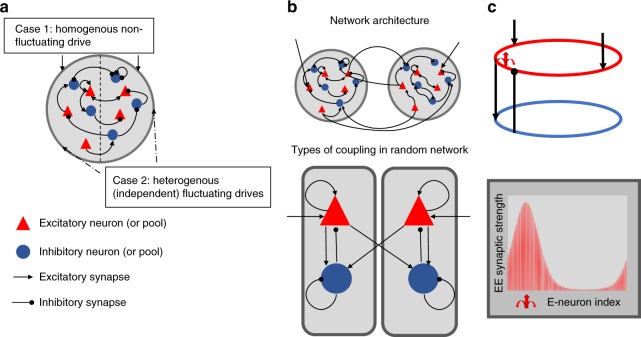
Three network architectures. **a** Unstructured network receives either a homogenous nonfluctuating drive (Case 1) or each half of the network receives independent stochastic (fluctuating) drive (heterogeneous drive, Case 2). **b** Discrete mutual-inhibition network consists of two pools of excitatory and inhibitory neurons tuned to a specific percept. The architecture of each pool is an unstructured network as in **a** and receives nonfluctuating drive to excitatory neurons only. Mutual inhibition model consists of long-range connections from excitatory neurons in one pool to inhibitory neurons in the other. **c** Continuum network consists of neurons arranged in excitatory and inhibitory rings with spatially structured coupling between all neuron types. Two sets of excitatory neurons on opposite sides of the ring (representing different percept tuning) receive nonfluctuating feedforward drive. Red arrow shows example of the spatial profile for excitatory-to-excitatory synaptic strength from a single presynaptic neuron. Strength is periodic and maximal at the presynaptic neuron (at red arrow)

**Fig. 2:**
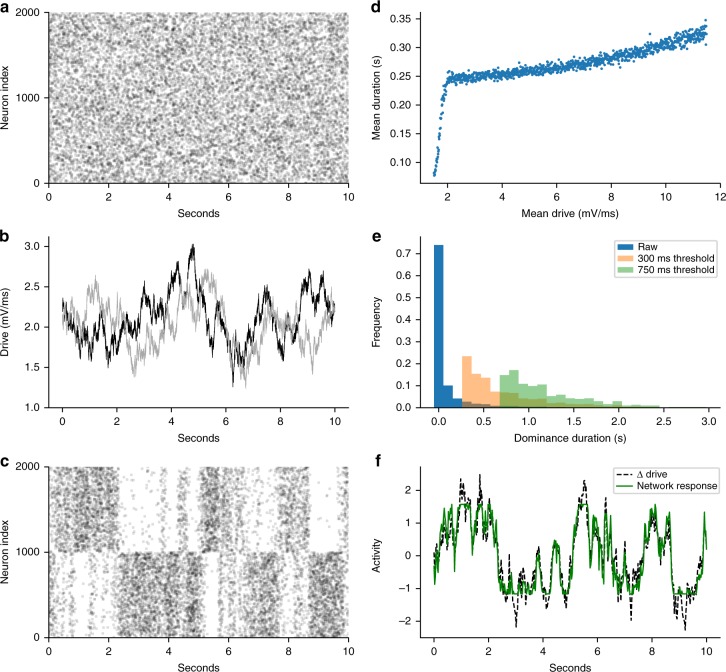
Unstructured network does not comply with rivalry constraints. **a** Case 1: homogenous drive raster (excitatory neuron spiking across time) exhibits homogenous response. Case 2: heterogeneous drive (**b**–**f**) **b** heterogeneous drive consists of independent stochastic processes for subsets of neurons within the unstructured network. **c** Excitatory neuron raster showing alternating activity levels between two pools in response to the heterogeneous input in **b**. **d** Levelt’s 4th proposition is not obeyed: percept durations increase with drive strength instead of decreasing. **e** Dominance time distribution of random network (as in **c**). Dominance duration coefficient of variation (*CV*_D_) depends on the report threshold (see Table 1*:* definitions). For example, *CV*_D_ are 1.9, 0.61, and 0.34 for 0, 300, and 750 ms report thresholds, respectively. **f** Percept state variable (*z*-scored) reflects differences in the drives in **b**, supporting that the dominance duration statistics mirror the drive fluctuations

We evaluated competition dynamics in an unstructured network (Fig. 1a) with balanced state dynamics and tested if it could match the empirical constraints of rivalry (see Table 1). Homogenous drive resulted in biophysical spiking and statistically homogenous irregular activity as predicted by the balanced state theory (Fig. 2a)^8,9^. The addition of a fatigue mechanism did not rescue the model (see Supplementary Information: Adaptation effect in the unstructured network). If subsets of neurons receive heterogeneous drive (Fig. 2b) then competition between two pools can emerge. This has been shown previously and associated with a (perceptual) decision making, choice task^33^, and thus applicable to our investigation. We found that the model resulted in epochs where one pool had a higher spiking rate than the other (Fig. 2c); however, the model failed to capture Levelt’s 4th proposition (Fig. 2d). In addition, the dominance duration distributions were not stable to changes in the report threshold (Fig. 2e). As shown in Fig. 2f, the competitive dynamics in the unstructured network mirrored the external drive^33^. The tight match between the drive and the network activity demonstrates that alternations resulted from the network activity tracking and amplifying differences in the feedforward drive. Thus this unstructured network does not generate rivalry dynamics itself. It could satisfy all empirical constraints if it received inputs from a rivalry source that satisfied the perceptual constraints if not the spiking constraints. However, this leaves the original source of the rivalry dynamics unexplained.

**Table 1:** Definitions and experimental constraints

Definitions
Population—set of neurons in a synaptic class, i.e., a set of excitatory or inhibitory neurons
Pool—a network of excitatory and inhibitory neurons tuned to a percept
Dominant pool—actively spiking neuronal pool (determined by the excitatory population activity), which corresponds to dominant percept
Suppressed pool—weakly active or inactive neuronal pool corresponding to suppressed percept
Dominance duration—time duration dominant pool remains highly active
Report threshold—minimum reportable event duration
Drive—external feedforward current (mV ms^−1^)
Experimental constraints
Perceptual constraints—Levelt’s proposition^56^:
1. Mean dominance duration is on the order of seconds.
2. Levelt’s 4th proposition—mean percept duration decreases with increased drive
3. Classical Levelt’s 2nd proposition—starting from the point where the stimulus drive gives equal dominance durations across the pools (equi-dominance), then weakening drive to one pool increases the opposite pool’s predominance
4. Modified Levelt’s 2nd proposition—from the equi-dominance point, strengthening drive to one pool increases the same pool’s predominance
5. Maximal alternation rate—when modifying drive to one pool, the rivalry alternation rate is fastest when both pools have equal dominance times^41^
Variability constraints:
Perceptual variability:
1. Dominance-time durations follow a gamma distribution with coefficient of variation (*CV*_D_) between 0.4 and 0.8^28^
2. Dominance statistics are robust to chosen report threshold
3. Mode of dominance time distribution is to the right of the report threshold
Spiking variability:
1. Mean spike-count correlations less than 0.3^57^
2. Spike count Fano Factor is between 0.9 and 2.0^4^
3. Coefficient of variation for interspike intervals is between 0.5^3^ and 2^7^ (target of 1.0 used for model fitting)
4. Mean spike-rates are between 5 and 40 Hz during upstates, and below 10 Hz when suppressed^4^

### Mutual inhibition networks can satisfy all constraints of rivalry

**Fig. 3:**
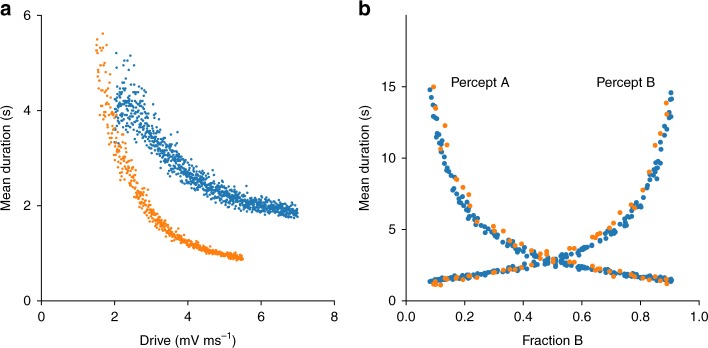
Psychophysics reproduced by discrete (blue) mutual inhibition and continuum (orange) networks. **a** Networks match Levelt’s 4th proposition; dominance duration decreases with drive strength. **b** Networks match Levelt’s 2nd and modified 2nd propositions (compare with Fig. 6 in ref. ^41^). Consistent with this, the overall alternation rate decays symmetrically from equal dominance

**Fig. 4:**
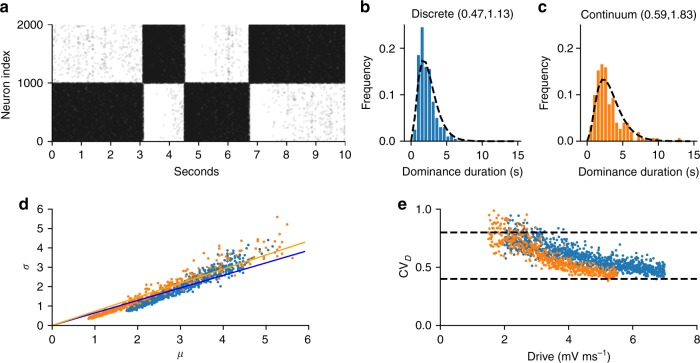
Psychophysics variability reproduced by discrete (blue) mutual inhibition and continuum (orange) networks. **a** Excitatory neuron raster of discrete mutual inhibition network during rivalry showing stochastic dominance durations. **b**, **c** Gamma-like distribution of dominance durations with 300 ms report threshold (see Table 1). *CV*_D_ and skewness, respectively, are given in parenthesis. Dashed lines in **b**, **c** are the empirical gamma distribution shape parameter from Robertson et al. during binocular rivalry^42^ using the scale parameter from our simulations. **d** Dominance duration standard deviation (*σ*) vs mean (μ) is well fit by regression line with slope *CV*_*D*_ matching Cao et al.^28^ (discrete slope = 0.65, *P*-value < 10^−314^, *n* = 999 drive strengths sampled; continuum slope = 0.73, *P*-value < 10^−314^, *n* = 500 drive strengths sampled). **e** *CV*_*D*_ computed at each drive strength stays within the experimentally observed range (dashed lines) across changes in drive but has a significant trend (*P*-value < 10^−159^, same samples as in **d**). Statistics (two-tailed *P*-values, etc.) for descriptive purpose of the modeled system and not rigorous hypothesis tests

**Fig. 5:**
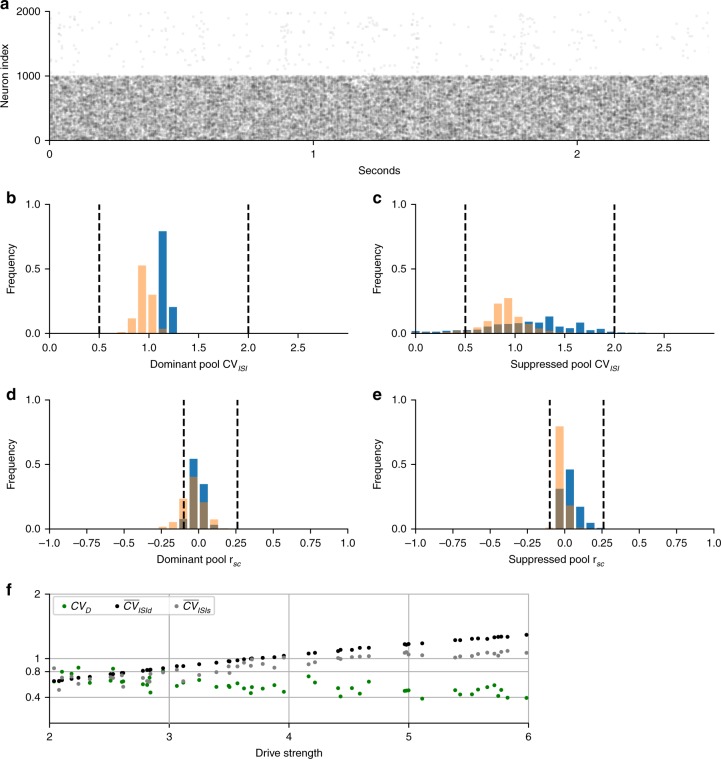
Spiking variability in discrete mutual inhibition (blue) and continuum (orange) networks. **a**–**e** Results from single realization of example systems (symmetric drive = 5). Distributions are across neurons and dashed lines are empirically observed bounds (see Table 1). **a** Excitatory neuron raster of discrete mutual inhibition network during rivalry showing stochastic spiking in dominant (neuron index 0–999) and suppressed (neuron index 1000–2000) populations. **b**, **c** Irregular spiking shown by the interspike-interval coefficient of variation (CV_ISI_) distributions for dominant pool (**b**) and suppressed pool (**c**) excitatory neurons. **d**, **e** Asynchronous spiking of dominant pool (**d**) and suppressed pool (**e**) excitatory neurons shown by the distribution of spike-count correlations (r_sc_). **f** Spiking and perceptual variability as a function of drive strength obeying Levelt’s 4th proposition. Plot of CV_D_ and the average CV_ISI_ across neurons in the dominant $\left( {\overline {CV} _{{\mathrm{ISId}}}} \right)$ or suppressed $\left( {\overline {CV} _{{\mathrm{ISIs}}}} \right)$ states. There was a significant linear trend for all measures (maximum *P*-value < 10^−8^, *n* = 49 drive strength samples). Measures remained within the empirical constraints as indicated by y-ticks. Statistics (two-tailed *P*-values, etc.) for descriptive purpose of the modeled system and not rigorous hypothesis tests

We evaluated two mutual inhibition network architectures. One was a discrete mutual inhibition network that was previously used to model rivalry, flanker-suppressor tasks, and normalization^19^ (Fig. 1b). The second was a modification of a continuum network used previously for rivalry and other tasks^20,29^ (Fig. 1c). Neither of these systems had been shown to satisfy all of the empirical constraints in Table 1. We numerically scanned parameter space in both architectures to find regions where the rivalry constraints were satisfied. The networks were completely deterministic and received constant nonfluctuating drive. Figures 3–5 show results for single example models of each network architecture (discrete and continuum) that matched the constraints, where for each model all parameters except drive were fixed. A summary of matched perceptual constraints is shown in Fig. 3, perceptual variability results in Fig. 4, and spiking variability in Fig. 5. In Figs. 3 and 4, points are multiple realizations across drive strengths for the example models. Results for Fig. 5a–e are for a single realization and 5f for multiple realizations of the models.

The models matched all of the Levelt’s proposition constraints. As shown in Fig. 3a, the dominance times decreased with strength in both models, in keeping with Levelt’s 4th proposition. In response to asymmetric drive strengths (Fig. 3b), both models matched the characteristic dominance duration profiles seen in ref. ^41^ and produced the characteristic ‘X’ shape of the classical and modified Levelt’s 2nd proposition. The alternation rate was maximal when both populations have the same dominance duration as expected.

Perceptual variability constraints could be satisfied by both models while still satisfying Levelt’s propositions. Dominance durations were irregular (see Fig. 4a) and showed gamma-like statistics. The distributions (see Fig. 4b, c) automatically matched the shape parameter from experiments on real subjects^42^ (indicated by dashed lines). (The shape parameter was not part of the model fitting scheme.) Fig. 4d shows that a plot of standard deviation vs mean of dominance duration is well fit by a regression line with the empirically observed *CV*_D_ slope^28^. However, when *CV*_D_ is computed independently for each drive strength, it shows a small but significant decreasing trend with increasing drive strength while staying within the empirical range (Fig. 4e). This has not been reported by others thus far. This small trend could serve as a falsifiable prediction for the models. In addition to *CV*_D_, Cao et al. showed that for many rivalry conditions the skewness of the dominance durations is within 1–4 times the *CV*_D_. We did not constrain skewness but *posthoc* discovered that our examples fell within this range providing an independent validation of the models. The average skewness/*CV*_D_ was 3 and 2.5 for the discrete and continuum models, respectively. This is higher than the twofold scaling suggested by Cao et al. but within the empirical range they reported. In addition, the histograms closely matched empirical parameters from Robertson et al. (Fig. 4b, c).

In addition to matching percept variability, the networks exhibited biophysical spiking (Fig. 5a). The example models exhibited irregular (Fig. 5b, c) and asynchronous (Fig. 5d, e) spiking. The average interspike-interval coefficient of variation *CV*_ISI_ was 1.22 during dominant states and 1.19 during the suppressed states. The average spike-to-spike correlations *r*_sc_ was 0.02 during dominant states, and 0.06 during suppressed states. All model spiking statistics fell well within the empirically reported ranges (indicated by dashed lines). As shown in Fig. 5f, we found a relationship between drive and spiking variability but in the opposite direction of *CV*_D_. Increasing drive strength decreased perceptual variability but increased spiking variability, when Levelt’s 4th proposition is obeyed. This forms an empirical prediction and test of the model during realistic stimulus conditions. In the next section we examine the underlying mechanism for the spiking variability and whether it conforms to balanced state theory.

Overall it was easier to find parameters that matched experimental constraints in the discrete versus the continuum model. For the discrete model, rivalry dynamics were found by starting with intra-pool parameters that led to biophysical spiking in each pool, then adjusting mutual inhibition strength until WTA appears. We then adjusted the adaptation strength and time constant to achieve rivalrous alternations. Since adaptation can affect spiking variability, parameters often had to be readjusted to recover biophysical spiking. Once both spiking and perceptual variability converged to the empirical constraints then, remarkably, rivalry dynamics satisfying all empirical constraints naturally emerged. The continuum model was harder to match since Gaussian footprints overlap and need to simultaneously satisfy both local and global dynamics. We used a random sampling approach to find conditions that matched all constraints since the effect of the parameters on local and global dynamics were not easily untangled. In numerical experiments, we found that in the continuum model, parameters for WTA were about tenfold more difficult to locate than for biophysical spiking, and that the combination of the two was rare but not overly difficult to find (see Supplementary Information: Continuum model parameter search).

### State dependent mechanisms for spiking variability

The results above demonstrate that the fully deterministic coupled network can support irregular neuron spiking in the presence of rivalry. Here we examine whether this variability is quantitatively explained by the balanced state theory of van Vreeswijk and Sompolinsky^8,9^. Balanced state theory argues that an attractor state for irregular spiking can exist in a network of excitatory and inhibitory neurons where the net mean input to each neuron in the network is balanced near the threshold of spiking and nonperiodic firing is self-consistently supported by the fluctuations in the input. The theory shows that this balanced state is maintained in the absence of any fine tuning of the coupling weights. Ingeniously, the theory demonstrates that regardless of the inherent nonlinearities of the intrinsic neuron dynamics, the balanced state attractor can be completely determined by the solutions of a purely linear system. Given that rivalry depends on mutual inhibition to allow for the suppression of one pool by another, it is not *a priori* certain that the balanced state theory would be directly applicable for rivalry.

We first consider the discrete architecture of a coupled network of four excitatory and inhibitory populations organized into two pools but in the absence of fatigue. Following the prescription of balanced state theory, we represent the state of the network by the mean spiking rates of each population, *r*_e1_, *r*_i1_, *r*_e2_, *r*_i2_, and the mean external drive to each population, *f*_e1_, *f*_i1_, *f*_e2_, *f*_i2_. We then suppose that the input to a neuron in one population from another population is proportional to the firing rate of the input population weighted by the mean coupling weight between the two populations. The total input to a population is then given by the sum over all the inputs from the other populations and the external drive. For example, the input to population e1 is *w*_e1e1_*r*_e1_ − *w*_e1i1_*r*_i1_ + *w*_e1e2_*r*_e2_ − *w*_e1i2_*r*_i2_ + *f*_e1_.

The balanced state for any set of coupling weights and external drives is given by the set of rates such that the mean input to every population is at threshold (in the mean field limit). This condition can be written in matrix form as **Wr** + **f** = 0, where **r** is the vector of spiking rates for each population, **f** is the vector of external inputs, and **W** is the 4 × 4 matrix of coupling weights between each population. A unique solution for **r** exists if and only if **f** is in the column space of **W**. This immediately shows that if the two pools are symmetrically coupled (i.e., *w*_e1e1_ = *w*_e1e2_ = *w*_e2e1_ = *w*_e2e2_, etc.) then a solution cannot exist if the external drive is not symmetric between the two pools (as in our unstructured network with heterogeneous drive and noted in ref. ^43^). Thus, a symmetrically coupled unstructured network cannot support a global balanced state. However, a balanced state can be supported if the symmetry is broken. In our structured discrete network, each individual pool is identically connected but the two pools are connected only through excitatory-to- inhibitory connections. The matrix equation has the form1

The sparse matrix allows symmetry between the pool rates to be broken and a solution for **r** is possible. Details of all calculations are given in the Supplementary Information: Balanced state theory. When tested whether this *mutual inhibition balanced state theory* explains the spiking variability of our simulation and as we show below, it depends on the dynamical (psychophysical) state of the system.

**Fig. 6:**
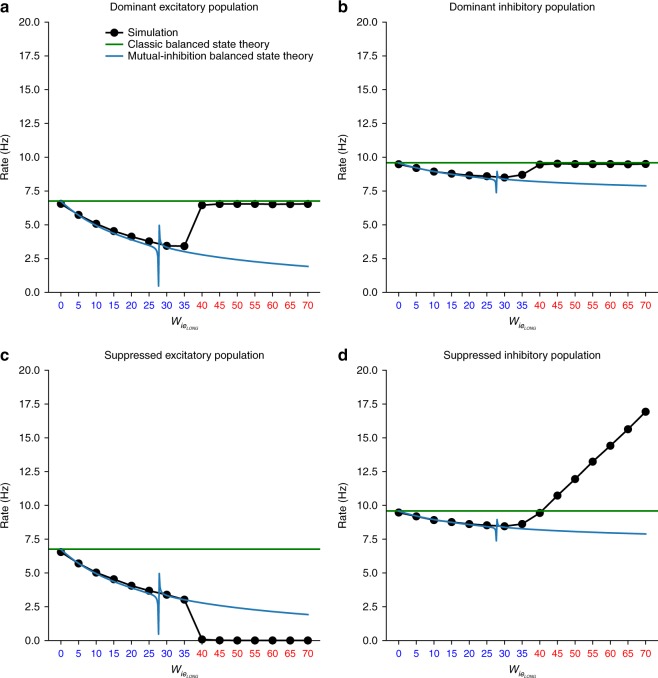
Transition from balanced to mixed balanced state across psychophysical states: **a** dominant pool excitatory population, **b** dominant pool inhibitory population, **c** suppressed pool excitatory population, and **d** suppressed pool inhibitory population. Black dots are simulation results. Blue lines are the two-pool mutual inhibition (four population) balanced state theory predictions. Green lines are the classic, single-pool (two population), balanced state theory predictions. Blue abscissa tick marks indicate the symmetric state and red indicate asymmetric state. Irregular spiking in the symmetric state is explained by mutual-inhibition balanced state theory but only the dominant state in the asymmetric state is explained by classic balanced state theory. The suppressed pool does not conform to balanced state theory although it still fires irregularly due to irregular input from dominant pool. Transition from symmetric to asymmetric state is anticipated by a singularity in the theory indicated by the discontinuity in the blue lines

We examined the applicability of the balanced state theory (classical single-pool theory and the two-pool mutual inhibition theory) to the neuronal network simulations by quantitatively comparing the predicted spiking rates, **r**, from the theory to the actual rates from the spiking network. Figure 6 shows the firing rates of all four populations as a function of the mutual inhibition weight between pools, $w_{{\mathrm{ie}}_{{\mathrm{LONG}}}}$. For low $w_{{\mathrm{ie}}_{{\mathrm{LONG}}}}$ the network is in a symmetric state where both pools have the same rates. The predictions of the mutual-inhibition balanced state theory (blue lines) match the simulated rates (black dots). The rates of the excitatory populations fall at a faster rate with $w_{{\mathrm{ie}}_{{\mathrm{LONG}}}}$ than the inhibitory populations as predicted by the theory and also consistent with a state of *normalization*^19^ where the mutual inhibition induces a sublinear response to inputs. Thus, irregular spiking in a balanced state can coexist with a psychophysical state of normalization.

As $w_{{\mathrm{ie}}_{{\mathrm{LONG}}}}$ is further increased, the symmetric normalization state makes a transition to an asymmetric WTA state^19^ (a precursor for rivalry) where the excitatory neurons of the dominant pool has higher activity than the suppressed pool (Fig. 6a, c) and vice versa for the inhibitory populations (Fig. 6b, d). After this transition, the mutual inhibition theory no longer matches the simulations. However, if we consider the pools to be uncoupled then the uncoupled single-pool theory (green line), which corresponds to the classic balanced state solution^8,9^, matches the firing rate of the dominant pool but not the suppressed pool. The dominant pool is effectively uncoupled because the input from the suppressed pool is negligible while the suppressed pool is not in a balanced state because the local recurrent excitation cannot balance the inhibitory input from the dominant pool. This can be taken to represent the converse to the scenario of Ebsch and Rosenbaum. Although not in a balanced state, the suppressed pool still fires irregularly and asynchronously (as shown in Fig. 5) because it is driven by the balanced irregularly spiking dominant pool. The symmetric to asymmetric state transition was actually anticipated by the appearance of a singularity in the balanced state theory solutions at a critical value of $w_{{\mathrm{ie}}_{{\mathrm{LONG}}}}$ (indicated by the discontinuity in the blue lines in Fig. 6). The singularity represented a breakdown of the mutual inhibition balanced state theory and this was borne out by the state transition in the simulations.

The above results will still apply in the presence of neuronal adaptation and hence rivalry if the adaptation time constant is slow. The effect will be equivalent to adiabatically changing the external drive. In the symmetric state, this will simply weaken the external drive and the system will settle into a new symmetric state with lower rates while preserving irregular spiking due to the balanced state. In the asymmetric state, the dominant pool will be balanced while the suppressed state will not. The adaptation will relax in the suppressed pool until the net input is sufficiently strong to overcome the inhibition from the dominant pool and cause a switch in dominance, whereupon the newly dominant pool will be in a balanced state while the newly suppressed pool will not.

## Discussion

We asked if psychophysical responses (specifically rivalry) and the balanced state can coexist in a single canonical circuit model, and thus provide a self-consistent model for spiking and perceptual variability. We found that the answer is yes and no. Indeed, spiking and perceptual variability (and other psychophysical phenomena) are explained by the same deterministic system. However, further investigation of the balanced state theory reveals a deviation from pure balance. The two-pool mutual inhibition balanced state theory we used is applicable to WTA dynamics and cases where both percept-pools are active such as in normalization^19,44^. When we analyzed the mechanism for biophysical spiking and WTA, we found that the dynamics are due to a mixture of balanced and imbalanced states within the network. This gives an interesting twist to current notions of spiking in the brain. Irregular, asynchronous spiking may imply that the brain is either in a balanced state or it is being driven by a balanced state. In our case, mutual inhibition connections were sparse which maintained the variability in the drive to the imbalanced pool. Alternatively Darshan, van Vreeswijk, and Hansel^45^ showed how variability could be maintained in the more general case, which could be added to our model.

Our findings also augment our understanding of rivalry. Rivalry is an alternation in percept states that depends on a neuronal fatigue process such as spike-frequency adaptation or synaptic depression. During a dominance epoch, the dominant pool is above a neuronal threshold while the suppressed pool is subthreshold. There are two dynamical possibilities for how alternating dominance could occur. The first is called *release*, where a dominance switch occurs when neurons in the dominant pool fatigue to the point of falling below the spiking threshold and stop spiking. The second is *escape*, where neurons in the suppressed pool recover from fatigue and overcome the suppressing inhibition and thereby become dominant^46^. Levelt’s propositions require the escape mechanism^32^. This necessity is clearly seen in Levelt’s 4th proposition, which states that dominance duration increases with decreasing drive strength. A release mechanism would predict the opposite since decreasing drive would mean that a fatiguing neuron would drop below threshold faster and thus decrease dominance duration. However, under escape, a decreased drive would prolong the time of a suppressed neuron to recover from fatigue and spike and thus increase the dominance duration. The dominance duration is given by the time to escape, which is governed by the time constant of the fatigue mechanism (e.g., adaptation). However, the precise moment of escape is determined by the net input to the neuron as a function of time. If the neuron input is subject to uncertainty or noise then the time to escape can be stochastic and if the approach to threshold is shallow then even a small amount of noise can lead to a large amount of dominance duration variability. We show that self-induced variability of the balanced state in the dominant pool is sufficient to act as the noise source in the suppressed pool to explain perceptual rivalry. This is a biophysical realization of the Ehrenfest stochastic process proposed by Cao et al. for rivalry, where the stimulus dependent activation rate of the Ehrenfest process corresponds to the stimulus dependent escape rate of the suppressed population.

Further examination of our model, including different neuron models and architectures, is warranted to discover the extent and robustness of our findings. Our analyses were also restricted to the two-pool competition case. This represents the minimal competition model that explains many general rivalry effects. However, specific effects such as mixed perception in binocular rivalry may require three or more pools. The model can be expanded for these cases. We propose a refinement to a prior hypothesis for the invariance of perceptual variability across stimulus conditions. Cao et al. noted that perceptual variability (*CV*_D_) was near 0.6 across many competition conditions from binocular rivalry to motion-induced rivalry. This led them to hypothesize an invariance for rivalry variability. However, we found that increased drive strength can decrease the *CV*_D_; though, some parameter cases were more stable. This suggests that there may be finer structure within the experimental uncertainty observed in Cao et al. Similarly, we observed a spiking variability effect due to drive (when controlling for dominant or suppressed state), but in the opposite direction of perceptual variability. Notably this is within the stimulus range of behavioral experiments and forms an additional prediction of the model. It is paramount to test these model predictions in experiments to further refine the model. Further theoretical work is warranted to investigate the relationship between drive, scale, and other circuit parameters.

Does this model have utility and implications beyond rivalry? Rivalry stands out among perceptual phenomena since concrete changes in perception occur despite a static stimulus, thus providing insight into internal computations of the brain. It has even been considered a tool for investigating neural correlates of consciousness^47^. It is not surprising then that rivalry is one of the target behaviors of a developing canonical cortical circuit theory of cognition^21^. Similar circuit architectures can explain putative cognitive primitives such as short-term memory and decision making and have been used to interpret differences in cognitive measures among clinical, psychiatric cohorts^48–51^. A major challenge of psychiatric models is how to scale from the molecular perturbations underlying mental illness to complex psychopathology. Here we present a self-consistent theory for spiking and perceptual variability that bridges two important levels. However, this raises the question of the role of molecular variability, which exists in many forms^2^. It does not seem to be sufficient to explain spiking variability^9,33^ and, in light of our findings, it is unnecessary for a major form of perceptual variability. However, since the balanced state keeps neurons near threshold then small perturbations due to molecular noise could still have large effects. This will be interesting to study in the future. We propose that the influence of molecular variability is already contained within a set of hyperparameters of the cortical circuit governing the distribution of time constants and synaptic strengths.

Theories that tie together biology at multiple scales will be useful for making psychiatric and cognitive problems tractable. One application is the *excitation-inhibition (EI) imbalance hypothesis*^21,51,52^ in mental illness, a pervasive but ill-defined hypothesis in clinical research. Our model is based on two important EI relationships, the balanced state and mutual inhibition, and thus is a good candidate for exploring these questions. EI can be manipulated in the model to make clinical research predictions at multiple scales of behavior. The model can also be used as middle ground to map clinically associated molecular factors to the circuit parameters to make further predictions.

## Methods

### Model neurons

Neuronal dynamics were modeled as leaky integrate-and-fire neurons with adaptation that obey

2 $$\frac{{dv_i}}{{dt}} = f_i + s_i\left( t \right) - \frac{{v_i}}{{\tau _{\mathrm{m}}}} - \gamma a_i\left( t \right),v_i < \theta$$

3 $$v_i \to v_i - \theta ,v_i = \theta$$

4 $$\frac{{ds_i}}{{dt}} = - \frac{{s_i(t)}}{{\tau _{\mathrm{s}}}} + {\sum} {w_{ij}} \delta (t - t_{{\mathrm{spike}}}^j)$$

5 $$\frac{{da_i}}{{dt}} = - \frac{{a_i}}{{\tau _{\mathrm{a}}}} + \delta (t - t_{{\mathrm{spike}}}^i)$$

where *i*, *j* are neuron indices, *v* is neuron voltage, *f* is feedforward current drive, *τ*_m_ is the membrane time constant, *w* is the synaptic strength from neuron *j* to *i*, *s* is synapse strength, *τ*_s_ is the synaptic time constant, *a* is an adaptation variable, *γ* is the adaptation strength, *τ*_a_ is the adaptation time constant. The voltage threshold was 20 mV and the membrane and synaptic time constants were 20 and 2 ms, respectively. For the discrete and continuum case examples, the adaptation time constants were 350 and 650 ms and adaptation strengths were 0.44 and 0.013, respectively. We used a modified Euler’s method^53^ for simulations. In this scheme spike times (*t*_spike_) were interpolated where, at a given time step, any neuron that was above threshold was reset as its current voltage minus threshold. The time interval, *h*, was 0.1 and we verified that the dynamics were consistent with *h* = 0.01.

### Network architectures

We studied three randomly connected cortical circuit architectures (see Fig. 1): unstructured network, discrete mutual inhibition network, and structured continuum network. All network simulations had a total of 4,000 neurons. We checked to see that results did not qualitatively change with neuron number.

The *unstructured network* was evenly split between excitatory and inhibitory populations. Each neuron received *k* = 600 (in-synapses) randomly chosen connections for each neuron type (excitatory or inhibitory), with different synaptic strengths depending on the type of synapse. For example, an excitatory neuron received *k* excitatory and another *k* inhibitory synapses. The synaptic strengths for the example case were *A*_ee_ = 12.5, *A*_ie_ = 20, *A*_ei_ = 50, *A*_ii_ = 50 divided by the square-root of *k*, where *A*_ij_ is the synaptic strength from population *j* to *i*.

The *discrete mutual inhibition network* consisted of two unstructured-network pools as above, with 2000 neurons and *k* = 200 forming each percept pool. The two pools were also linked by *k* = 200 randomly chosen long-range connections from excitatory-to-inhibitory neurons ($A_{{\mathrm{ie}}_{{\mathrm{LONG}}}}$) (Fig. 1b top). In this case then, an inhibitory neuron not only received *k* excitatory and inhibitory synapses from within its pool but also *k* excitatory synapses from the competing pool. Synaptic strengths for the case example were *A*_ee_ = 10.5, *A*_ei_ = 20, *A*_ie_ = 30, $A_{{\mathrm{ie}}_{{\mathrm{LONG}}}}$ = 30, and *A*_ii_ = 45 divided by the square-root of *k*.

The *continuum network* consisted of 80% excitatory and 20% inhibitory neurons. Neurons were arranged evenly on a ring. They were connected with probability *p* and a synaptic strength obeying the von Mises distribution:

6 $$w_{ij}^C = \frac{{A^C{\mathrm{exp}}(\kappa ^C{\mathrm{cos}}(\theta _i - \theta _j))}}{{pN^ \ast 2\pi I_o(\kappa ^C)}}$$

where $w_{ij}^C$ is the synaptic weight for synapse class C (e.g., excitatory-to-excitatory, inhibitory-to-excitatory) from presynaptic neuron *j* to postsynaptic neuron *i*, *A*^*C*^ is a class dependent real amplitude, *κ*^*C*^ governs how fast the weights decayed, *N* is the number of postsynaptic neurons, *p* is the probability of a synaptic connection, *θ* is the neuron location in radians, and *I*_*o*_ is the modified Bessel function of order 0. For the case example, *κ*^*ee*^ = 0.26, *κ*^*ei*^ = 0.93, *κ*^*ie*^= 0.97, *κ*^*ee*^ = 0.5, *A*_ee_ = 84, *A*_ei_ = 314, *A*_ie_ = 1319, *A*_ii_ = 689, and *p* = 0.34.

### Drive

We manipulated feedforward drive to neurons in several ways. For the unstructured network we examined two cases: homogenous and heterogeneous drives. In the homogeneous case, excitatory (E) neurons received the same nonfluctuating drive that was slightly higher than the inhibitory (I) neurons. For the heterogeneous case, the network was divided into two excitatory pools each receiving an independent fluctuating drive, modeled as Ornstein–Uhlenbeck stochastic processes (mean for E = 0.2, mean for I = 0.1, *SD* = 1, *τ* = 500 ms). The OU time constant was chosen to obtain reasonable dominance durations. All structured network simulations received nonfluctuating drive to only the excitatory neurons. For the discrete mutual inhibition model, all excitatory neurons received a stimulus drive. For the continuum model, only a subset of excitatory neurons received this input. Levelt’s propositions were investigated by changing drive strengths as detailed in figures and computer code.

### Measures

Dominance durations were estimated by converting spike rates across pools into a *percept state variable P*, where *u*_A_ and *u*_B_ were the sum of spikes across excitatory neurons of each pool for 50ms time windows. In each window, the percept state was assigned to (*u*_A_ − *u*_B_)/(*u*_A_ + *u*_B_), which is a number between −1 and 1. We divided this domain into even thirds to classify the percept state: percept A (*P* > 1/3), percept B (*P* < −1/3), and neither percept A or B (−1/3 < *P* < 1/3). A dominance duration was measured as the interval between state changes. Spike count Fano factor and spike-count correlations were computed over 100 ms windows for each neuron. The interspike-interval coefficient of variation (*CV*_ISI_) was estimated for each neuron across simulation blocks. We isolated dominant vs suppressed states and calculated statistics for each state.

### Statistics and reproducibility

Figures 3d, e and 5f were fit with linear models for descriptive purposes. Significance is reported as two-tailed *P*-values. For Fig. 3d, e, *n* = 999 and 500 drive strength samples for the discrete and continuum models respectively, and for Fig. 5f, *n* = 49 drive strength samples.

### Reporting summary

Further information on research design is available in the Nature Research Reporting Summary linked to this article.

## Supplementary information

Supplementary Information

Reporting Summary

## Data Availability

Data used to generate the main figures^54^ are available via Figshare at 10.6084/m9.figshare.8869109.v1.

## Code availability

Computer code^55^ with detailed methods can be found at https://github.com/ShashaankV/multiscalebrainvar. Data can be generated using the published parameters here and in the code. Data used to generate figures^54^ are available via Figshare at 10.6084/m9.figshare.8869109.v1.
