# Supplementary material for: Dynamical modeling of multi-scale variability in neuronal competition
Source: Commun Biol. 2019 Aug 23;2:319. doi: 10.1038/s42003-019-0555-7 (PMC6707190; doi:10.1038/s42003-019-0555-7)
Supplement: Supplementary file 1 — Supplementary Information [file 42003_2019_555_MOESM1_ESM.pdf]

1. Adaptation effect in the unstructured network

Since random networks amplify asymmetries in their time-dependent feedforward inputs, we tested whether adding a neuronal fatigue process could result in oscillations when the inputs are constant but asymmetric. Drive parameters were $f_{\mathrm{e}1} = 3.0\frac{mV}{ms}, f_{\mathrm{e}2} = 3.5\frac{mV}{ms}, f_{\mathrm{i}1} = \frac{2.9mV}{ms}, f_{\mathrm{i}2} = 3.4\frac{mV}{ms}$. Coupling parameters were the same as used in main text. Strong adaptation was required to produce oscillations, with an amplitude of 5 $\frac{mV}{ms}$ and a time constant of 1 second. Oscillations were most pronounced when both excitatory and inhibitory neurons adapted (Supplementary Figure 1). Each excitatory population had oscillating activity levels, but this does not appear to reflect rivalry since each population appears to have an independent oscillatory frequency. This transient behavior disappeared entirely when the adaptation variable was initialized randomly across neurons.

Supplementary Figure 1

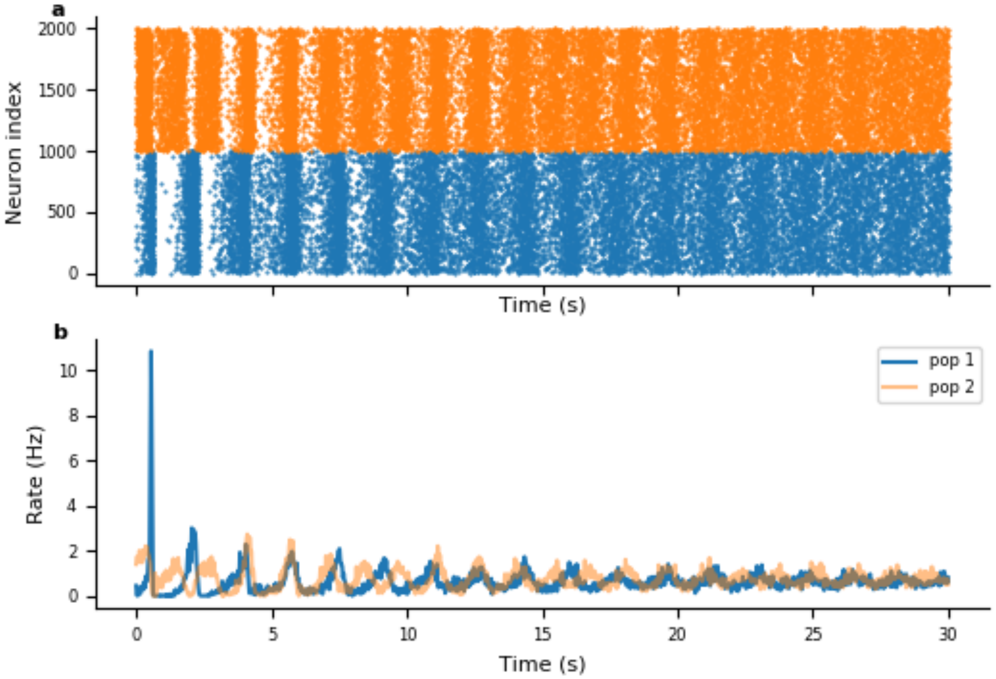

Supplementary Figure 1: Decaying oscillations in a random network with constant asymmetric drive and adaptation. A) Excitatory raster for two excitatory populations with different drive in the same network. **B)** Population averaged firing rates in contiguous 25ms time windows for each excitatory population.

2. Continuum model parameter search

In the continuum model, we randomly sampled synaptic coupling parameters without adaptation in a million networks each with 2,000 excitatory neurons and 500 inhibitory neurons. Out of the million simulations, 713,608 completed successfully. Failed simulations typically had excessive recurrent excitation which caused an inordinate number of spikes so that the simulation did not run to completion in the allotted time. Equations were solved using the forward Euler method, which we implemented using Julia version 0.4.5.

We classified networks according to the following (nonexclusive) dynamical properties: 1) *winner-take-all* (WTA) meaning that 90% of the total number of spikes were from one population, 2) *asynchronous* meaning that mean spike-count-correlations within a population were less than 0.1, and 3) *irregular* meaning that the spike count Fano factor was between 0.7 and 2.5. Of all models, 46.2% exhibited asynchronous and irregular spiking characteristics; whereas, only 3.9% of models exhibited winner-take-all behavior. Of winner-take-all models, 26.9% had asynchronous irregular spiking. Networks with both irregular and asynchronous spiking were deemed *biophysical*.

We sampled 2,000 models randomly from the collection exhibiting WTA and biophysical spiking, and scanned 1,000 pairs of adaptation parameters in each one. The adaptation time constant varied from 500ms to 5,000ms, and the amplitude varied from 0 to 0.05mV. To select reliable models, we isolated models with data for at least 20 simulations at different adaptation strengths. A small number of simulations would fail, leaving 1,996 reliable models. Of these, 1,949 exhibited rivalry with mean dominance time longer than 1 second in one population for at least one set of adaptation parameters. This showed that the majority of models with WTA produce rivalry when adaptation is introduced.

The effect of adaptation on dominance time and spiking statistics was slightly different for each model. The coefficient of variation of dominance times, mean dominance time, and spiking statistics can be affected simultaneously, so a successful model has adaptation parameters that align all three measures in realistic regimes. A parameter Venn diagram representation of models achieving success is shown in Supplementary Figure 2.

Supplementary Figure 2

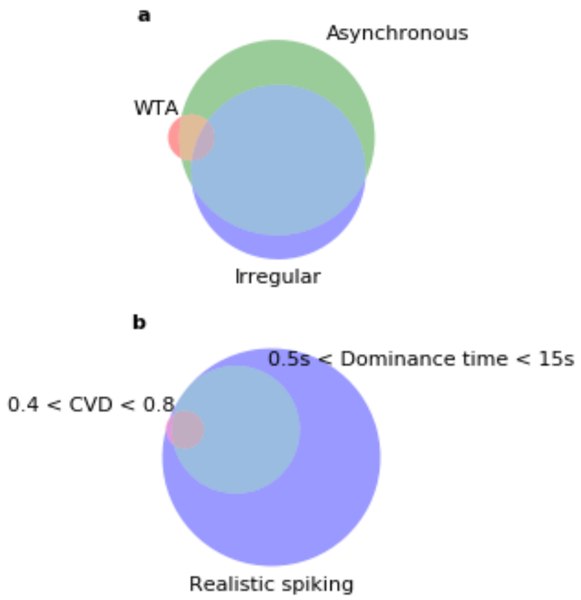

Supplementary Figure 2: Fractions of parameter space in continuum model satisfying various constraints. a) Arrangement of asynchronous, irregular, and WTA regimes in the 713, 608 models for which we had simulation data. **b)** A single model complying with all the constraints in (a) was selected and scanned for adaptation parameters. The arrangement of regimes with dominance times on the order of seconds, CV_D in the experimental range, and realistic spiking is shown.

3. Balanced-state theory

Consider first a random network of a single pool with an excitatory and an inhibitory population. We express the total inputs to neurons in terms of the spiking rates and coupling strengths of each population. Balanced-state theory argues that the excitatory and inhibitory inputs to neurons must balance near threshold in the limit as N goes to infinity to avoid a blowup of the spiking rates. This leads to a system of two equations – one for the input to the excitatory population, and one for the input to the inhibitory population:

$$I_{\mathrm{e}} = w_{\mathrm{ee}} r_{\mathrm{e}} - w_{\mathrm{ei}} r_{\mathrm{i}} + f_{\mathrm{e}} = 0 \tag{1}$$

$$I_{\mathrm{i}} = w_{\mathrm{ie}} r_{\mathrm{e}} - w_{\mathrm{ii}} r_{\mathrm{i}} + f_{\mathrm{i}} = 0 \tag{2}$$

where w_{xy} is the mean coupling strength from neurons in population *y* to neurons in population *x*. This can be written in matrix form as

$$\mathbf{Wr} + \mathbf{f} = \mathbf{0} \tag{3}$$

where

$$\mathbf{W} = \begin{bmatrix} w_{\mathrm{ee}} & w_{\mathrm{ei}} \\ w_{\mathrm{ie}} & w_{\mathrm{ii}} \end{bmatrix}, \mathbf{r} = \begin{bmatrix} r_{\mathrm{e}} \\ r_{\mathrm{i}} \end{bmatrix}, \text{ and } \mathbf{f} = \begin{bmatrix} f_{\mathrm{e}} \\ f_{\mathrm{i}} \end{bmatrix}$$

The firing rates of each population can be solved for if the external drives are in the column space of the coupling matrix $\mathbf{W}$.

This becomes an important issue if we arbitrarily divide the homogeneous random network into two pools of excitatory and inhibitory neurons and give each pool a different drive. The system can then be expressed using four populations:

$$\begin{bmatrix} w_{\mathrm{ee}} & w_{\mathrm{ei}} & w_{\mathrm{ee}} & w_{\mathrm{ei}} \\ w_{\mathrm{ie}} & w_{\mathrm{ii}} & w_{\mathrm{ie}} & w_{\mathrm{ii}} \\ w_{\mathrm{ee}} & w_{\mathrm{ei}} & w_{\mathrm{ee}} & w_{\mathrm{ei}} \\ w_{\mathrm{ie}} & w_{\mathrm{ii}} & w_{\mathrm{ei}} & w_{\mathrm{ii}} \end{bmatrix} \begin{bmatrix} r_{\mathrm{e1}} \\ r_{\mathrm{i1}} \\ r_{\mathrm{e2}} \\ r_{\mathrm{i2}} \end{bmatrix} = - \begin{bmatrix} f_{\mathrm{e1}} \\ f_{\mathrm{i1}} \\ f_{\mathrm{e2}} \\ f_{\mathrm{i2}} \end{bmatrix} \tag{4}$$

The only difference between the two pools are the drives f; the intra- and inter-pool coupling is identical. This imposed symmetry (note symmetry is between pools, coupling matrix need not be symmetric) makes the coupling matrix $\mathbf{W}$ rank 2. The column space is symmetric between the pools and thus solutions for the rates only exist if *f* is also symmetric (i.e. $f_{\mathrm{e1}} = f_{\mathrm{e2}}$, $f_{\mathrm{i1}} =$

f_{i2}). Therefore, the homogeneous random network driven by spatially inhomogeneous drive cannot obey the balance conditions *per se*.

We can break the pool symmetry if we assume that intra- and inter-pool connections have different weights such as a network with two mutually inhibiting pools. We assume each pool is identical and replace long-range connections with notation $w_{\mathrm{ie_{LONG}}}$. This leads to a system of four equations with four unknowns that obey

$$\begin{bmatrix} w_{\mathrm{ee}} & w_{\mathrm{ei}} & 0 & 0 \\ w_{\mathrm{ie}} & w_{\mathrm{ii}} & w_{\mathrm{ie_{LONG}}} & 0 \\ 0 & 0 & w_{\mathrm{ee}} & w_{\mathrm{ei}} \\ w_{\mathrm{ie_{LONG}}} & 0 & w_{\mathrm{ie}} & w_{\mathrm{ii}} \end{bmatrix} \begin{bmatrix} r_{\mathrm{e1}} \\ r_{\mathrm{i1}} \\ r_{\mathrm{e2}} \\ r_{\mathrm{i2}} \end{bmatrix} = - \begin{bmatrix} f_{\mathrm{e1}} \\ f_{\mathrm{i1}} \\ f_{\mathrm{e2}} \\ f_{\mathrm{i2}} \end{bmatrix} \tag{5}$$

This sparse matrix is full rank, so its columns span all possible input vectors *f.* Solving this system gives expressions for the firing rates of each pool. The expressions for the excitatory and inhibitory rates of pool 1 are

$$r_{\mathrm{e1}} = \frac{w_{\mathrm{ie_{LONG}}} f_{\mathrm{i2}} - \frac{w_{\mathrm{ie_{LONG}}} f_{\mathrm{e2}} w_{\mathrm{ii}}}{w_{\mathrm{ei}}} + (f_{\mathrm{i1}} - \frac{f_{\mathrm{e1}} w_{\mathrm{ii}}}{w_{\mathrm{ei}}})(\frac{w_{\mathrm{ee}} w_{\mathrm{ii}}}{w_{\mathrm{ei}}} - w_{\mathrm{ie}})}{\left(\frac{w_{\mathrm{ee}} w_{\mathrm{ii}}}{w_{\mathrm{ei}}} - w_{\mathrm{ie}}\right)^2 - {w_{\mathrm{ie_{LONG}}}}^2} \tag{6}$$

$$r_{\mathrm{i1}} = \frac{w_{\mathrm{ie}} r_{\mathrm{e1}} + w_{\mathrm{ie_{LONG}}} r_{\mathrm{e2}} + f_{\mathrm{i1}}}{w_{\mathrm{ii}}} \tag{7}$$

The expressions for pool 2 are the same with indices (1,2) exchanged. Note that when long-range connections are deleted, these expressions reduce to single-pool balanced state equations as expected.

Inputs balance to zero in units of threshold in this theory, but the threshold for an integrate-and-fire neuron in a network with a finite number of neurons is unknown. To account for this, we introduce variable threshold parameters T_{e} and T_{i} for excitatory and inhibitory neurons, which we subtract from the feedforward drive. The $\mathbf{W}$ parameters in our theory equations can be calculated analytically from our simulations parameters with

$$\mathbf{W}_{\mathrm{theory}} = \tau_{\mathrm{s}} k^{1/2} \mathbf{W}_{\mathrm{sim}} \tag{8}$$

This comes from the fact each neuron receives exactly *k* synaptic inputs of each connection type (excitatory or inhibitory), but individual synapses are scaled as $k^{-1/2}$. We do not normalize synaptic strength by the time constant so the time integral over the synaptic input will scale with the time constant. Alternatively, theory parameters can be calculated empirically by solving the equation

$$I_{ij} = w_{ij} r_j \tag{9}$$

By measuring the synaptic input from population *j* to population *i* and dividing by the firing rate of population *j* to recover theory parameters. We found that both methods return virtually identical results.

We first compared the single-pool balanced-state theory to simulations by perturbing w_{ei}. We found (see Supplementary Figure 3) that the predicted spiking rate trends from single-pool balanced state theory were similar to the simulations but spiking rates predicted from theory were systematically higher than we observed in simulations.

We next tested to see if whether a single value of T_{e} and T_{i} can account for the bias in the theory over the range of coupling parameters we tested. We searched this two-dimensional space for 100 values of each threshold parameter, spaced evenly from -3 to 3. For each set of thresholds, we compared theory predictions to observed rates and chose thresholds that minimized the summed Euclidean distance for excitatory and inhibitory neurons. This resulted in predictions that closely matched simulations within a reasonable range of coupling parameters

Supplementary Figure 3

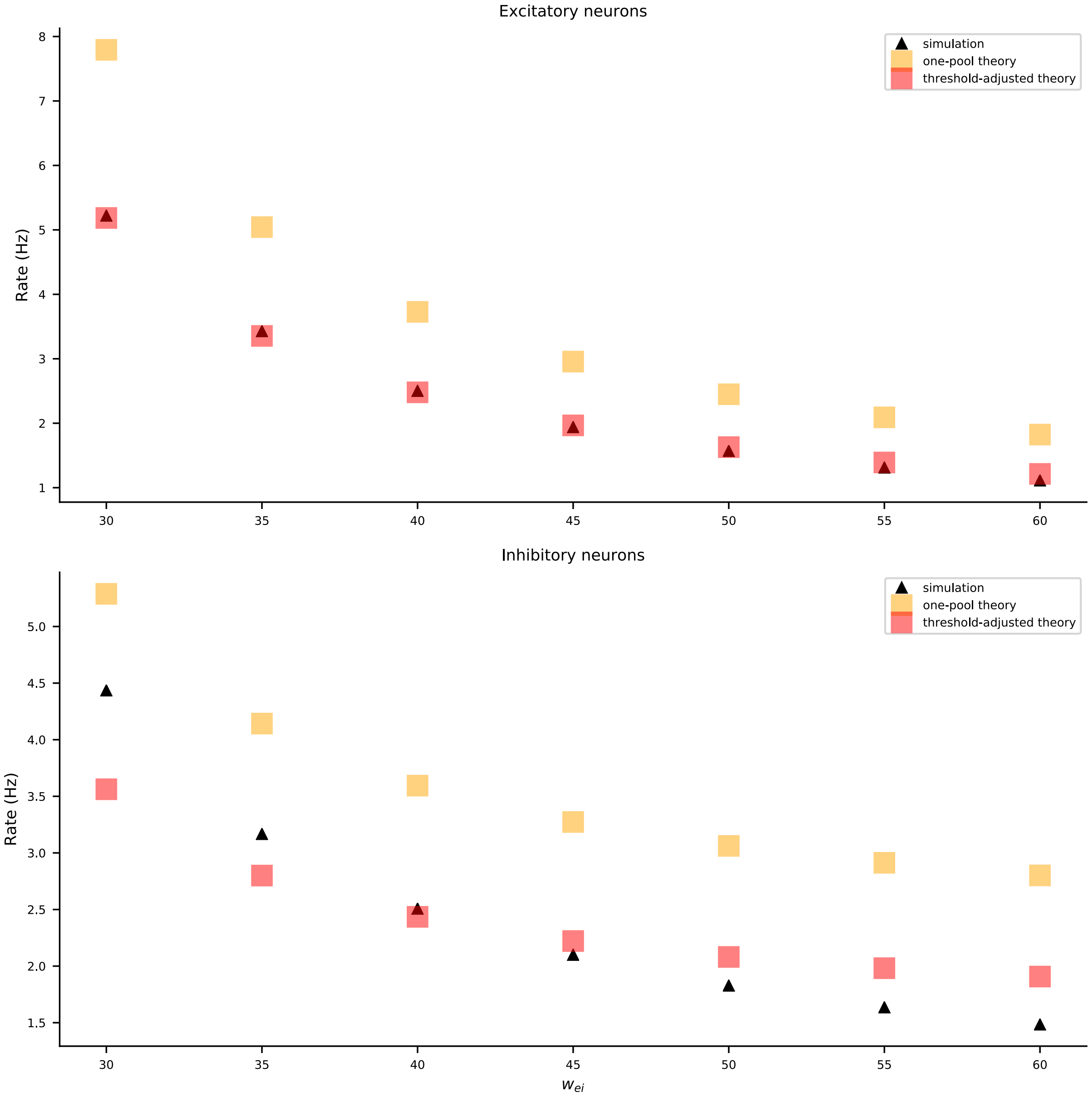

Supplementary Figure 3: comparison of single-pool balanced state theory to simulations. A) Excitatory firing rates in simulations and in theory predictions. **B)** Same as (A), but for inhibitory neurons.

With the capability to predict firing rates in single-pool networks of neurons, we turned to applying two-pool balanced state theory to mutual inhibition networks. These networks can exhibit WTA dynamics when mutual inhibition strength ($w_{\mathrm{ie}_{\mathrm{LONG}}}$) is strong enough. In order to apply two-pool theory to networks in the both-active state as well as the WTA state, we compared simulations to theory over a range of mutual inhibition strengths. We used the same process described above of recording rates from simulations, calculating theory parameters, scanning threshold parameters, and comparing theory predictions to our results.

In the both-active regime, two-pool balanced state theory with a threshold adjustment closely matches the firing rates seen in our simulations. As mutual inhibition strength was increased, we observed that the theory equations become singular and the theory breaks down (Supplementary Figure 4). The location of the singularity demarked the transition from both-active to WTA as shown in the main text. Specifically, the numerator and denominator of the rate equations become zero simultaneously. To make the singularity visible, we perturbed one of the f terms by a miniscule amount (0.01 mVms^{-1}). The numerator and denominator components of our equations are plotted to make this clear. In Figure 6 of the main text, this tiny perturbation in inputs was used for both theory and simulation. The same threshold adjustment that was optimal for the two-pool theory was used in the single-pool theory.

Supplementary Figure 4

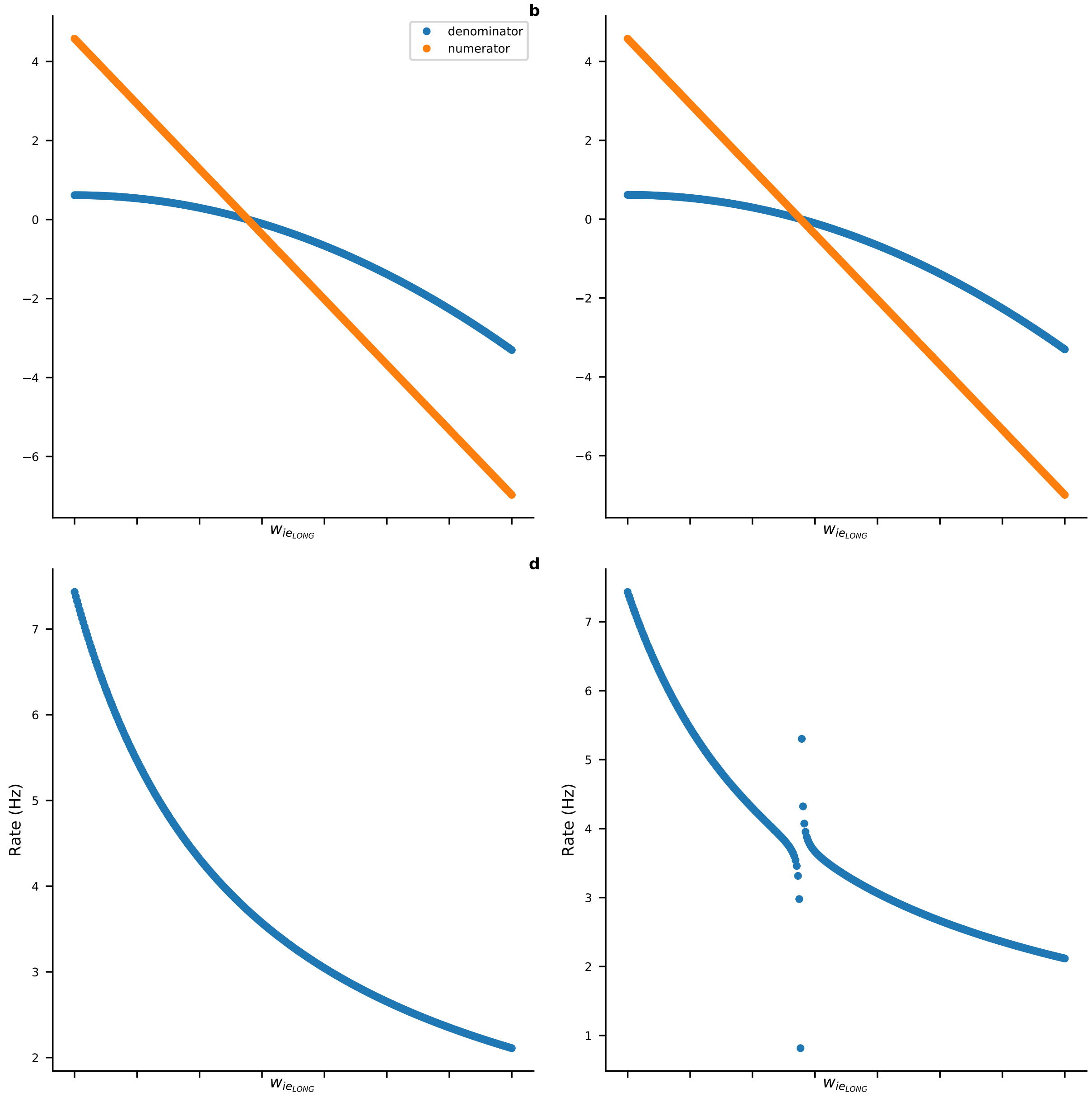

Supplementary Figure 4: Visualizing the singularity in the theoretical rate equations. **A)** Numerator and denominator of rate equations for various mutual inhibition strengths. Both cross 0 simultaneously. **B)** Same as (A), but with f_{e2} modified by 0.01. **C)** Rates predicted by theory. Singularity is not visible. **D)** Perturbing one of the drive terms makes numerator and denominator cross zero at slightly different locations, making the singularity obvious.
